# Preoperative imaging in primary hyperparathyroidism patients using 4DCT subtraction maps, a report of three cases

**DOI:** 10.1016/j.radcr.2023.05.019

**Published:** 2023-06-08

**Authors:** Jorian P. Krol, Tessa Veerbeek, Laura N. Deden, Frank B.M. Joosten, Cornelis H. Slump, Wim J.G. Oyen

**Affiliations:** a Department of Radiology & Nuclear Medicine, Rijnstate Hospital, Arnhem, The Netherlands; b Department of Robotics and Mechatronics, Faculty of Electrical Engineering, Mathematics and Computer Sciences, University of Twente, Enschede, The Netherlands; c Department of Biomedical Sciences and Humanitas Clinical and Research Centre, Department of Nuclear Medicine, Humanitas University, Milan, Italy; d Department of Radiology & Nuclear Medicine, Radboud University Medical Centre, Nijmegen, The Netherlands

**Keywords:** Primary hyperparathyroidism, Four dimensional computed tomography, Subtraction map, Parathyroid adenoma, Parathyroid hyperplasia

## Abstract

Four-dimensional computed tomography (4DCT) is one of the preoperative imaging modalities that can be used to localize a parathyroid adenoma in primary hyperparathyroidism patients however, sensitivity differs in literature and could be improved especially for multiglandular hyperplasia or double adenomas. The most robust feature on the 4DCT for the differentiation between parathyroid adenoma and thyroid gland tissue is arterial enhancement. To make this better visible, we have developed a subtraction map that shows arterial enhancement as a color scale to increase sensitivity for 4DCT. In this report of 3 cases, we present the usefulness of this subtraction map in a 54-year-old male, a 57-year-old female and a 51-year-old male. Subtraction maps may increase sensitivity for 4DCT, especially for multiglandular hyperplasia or double adenomas.

Primary hyperparathyroidism (PHPT) is caused by excessive secretion of parathyroid hormone, resulting in hypercalcemia [1]. PHPT is the third most common endocrine disorder after diabetes and thyroid diseases. The prevalence is between 0.1% and 0.7% in adults, 3 times more common in women than in men [2], [3], [4], [5], [6], [7]. In 80% of the cases, PHPT is caused by a single adenoma, followed by multiglandular disease in 15% and double adenomas in 4%-5% [1]. Parathyroid carcinoma is a very rare cause of hyperparathyroidism, in less than 1% of patients [8].

Historically, bilateral neck exploration (BNE) was the treatment of choice, without any prior imaging. However, with the introduction of minimally invasive parathyroidectomy (MIP), preoperative imaging has become essential to identify the location of the adenoma before surgery. Several imaging modalities can be used to localize a parathyroid adenoma, including 4-dimensional computed tomography (4DCT). The sensitivity of 4DCT ranges between 48% and 97% [9], [10], [11]. In literature, various imaging protocols are reported, but 4DCT normally consists of at least a nonenhanced phase, an arterial phase and a venous phase. In some protocols, a delayed venous phase is added. The 4DCT protocol in our institution consists of pre-contrast phased followed by injection of 90-120 mL iodine-based intravenous contrast agent (Xenetix 300, Guerbet, Villepinte, France). Arterial, venous and late venous phase were acquired with post-threshold delays of 10, 40, and 85 seconds, respectively. The various phases of 4DCT are used to identify the parathyroid adenoma. The parathyroid adenoma has a lower attenuation on the nonenhanced phase as compared to the thyroid gland, reflected by a lower Hounsfield Unit (HU) value. The parathyroid adenoma is highly vascularized and therefore shows a characteristic pattern of enhancement. This manifests in rapid contrast wash-in as compared to the thyroid gland on the arterial phase, and increased contrast wash out in the venous and delayed phase. However, Bahl et al. showed 3 different attenuation patterns of the parathyroid adenoma relative to the thyroid gland. Parathyroid adenomas showed either higher attenuation than thyroid on arterial phase (type A), not higher attenuation on arterial phase but lower attenuation on venous phase (type B) or neither higher attenuation on arterial phase and lower attenuation on venous phase (type C) [12]. Furthermore, because of the embryological origin in the third and fourth parapharyngeal pouch for the inferior and superior parathyroid glands respectively, the anatomical location of the parathyroid gland can vary greatly. These parathyroid adenomas outside region the thyroid are referred to as ectopic and their location can be anywhere between the mandibular angle and the aortic arch in the mediastinum [13]. The adenoma's small size, the heterogeneity of attenuation patterns, and location complicate preoperative localization.

The arterial enhancement is the most robust feature present on the 4DCT to differentiate between parathyroid adenomas and thyroid tissue. To make this better visible, we have developed a subtraction map that shows the arterial enhancement as a color scale. In this report of 3 cases, we present the usefulness of this subtraction map. This report of 3 cases was approved by the local ethics review board (Reference number 2022-2154).

## Case 1

**Fig. 1:**
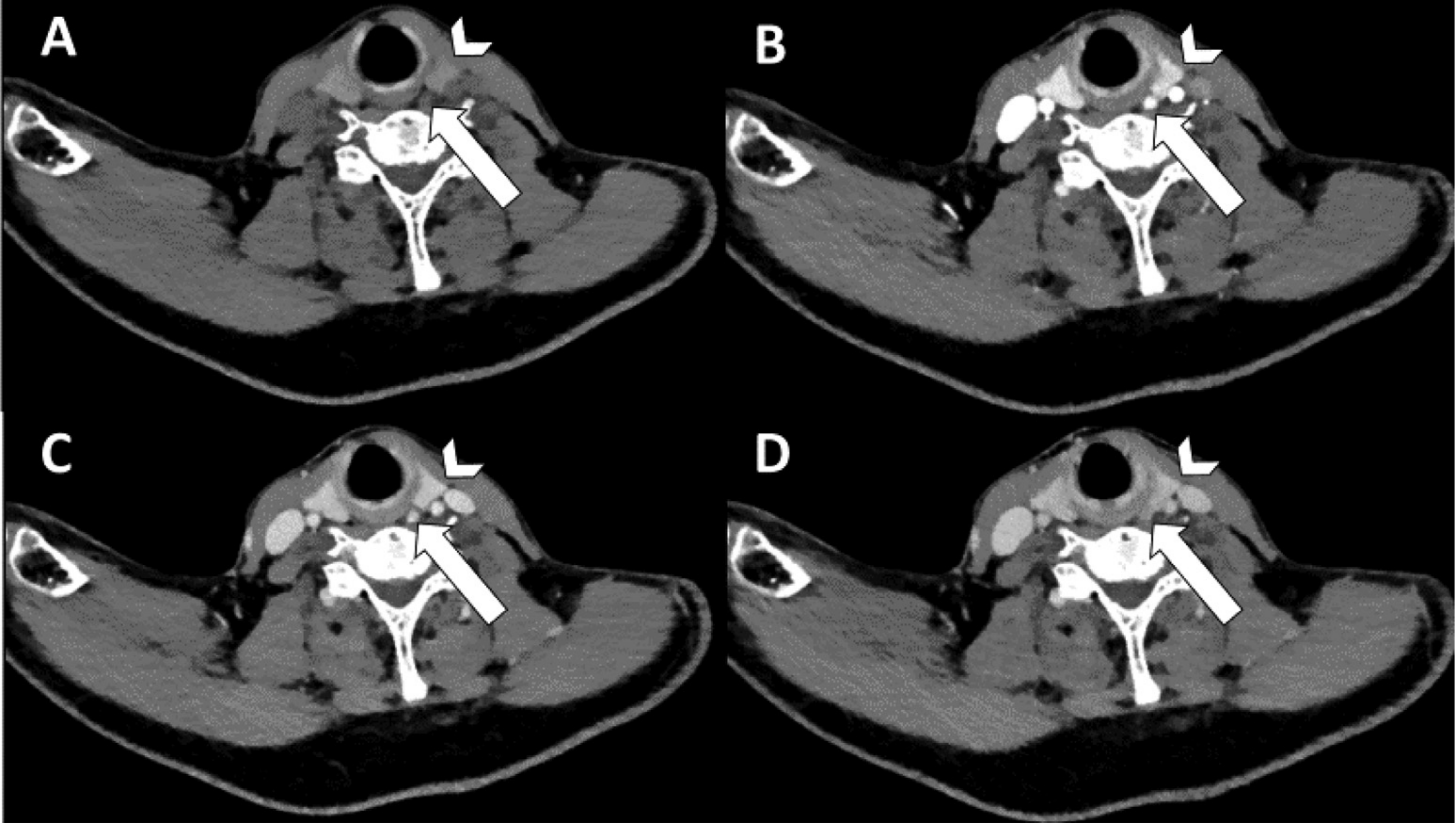
Four-dimensional CT with nonenhanced (A), arterial (B), venous (C) and delayed venous (D) phase of patient 1. The arrowhead is the thyroid gland. The arrow points to a lesion with lower attenuation than the thyroid gland on nonenhanced phase, with arterial enhancement and washout on the arterial and venous/delayed venous phases respectively. During surgery, after removing this lesion, PTH level did not decrease adequately, so further neck exploration was necessary.

**Fig. 2:**
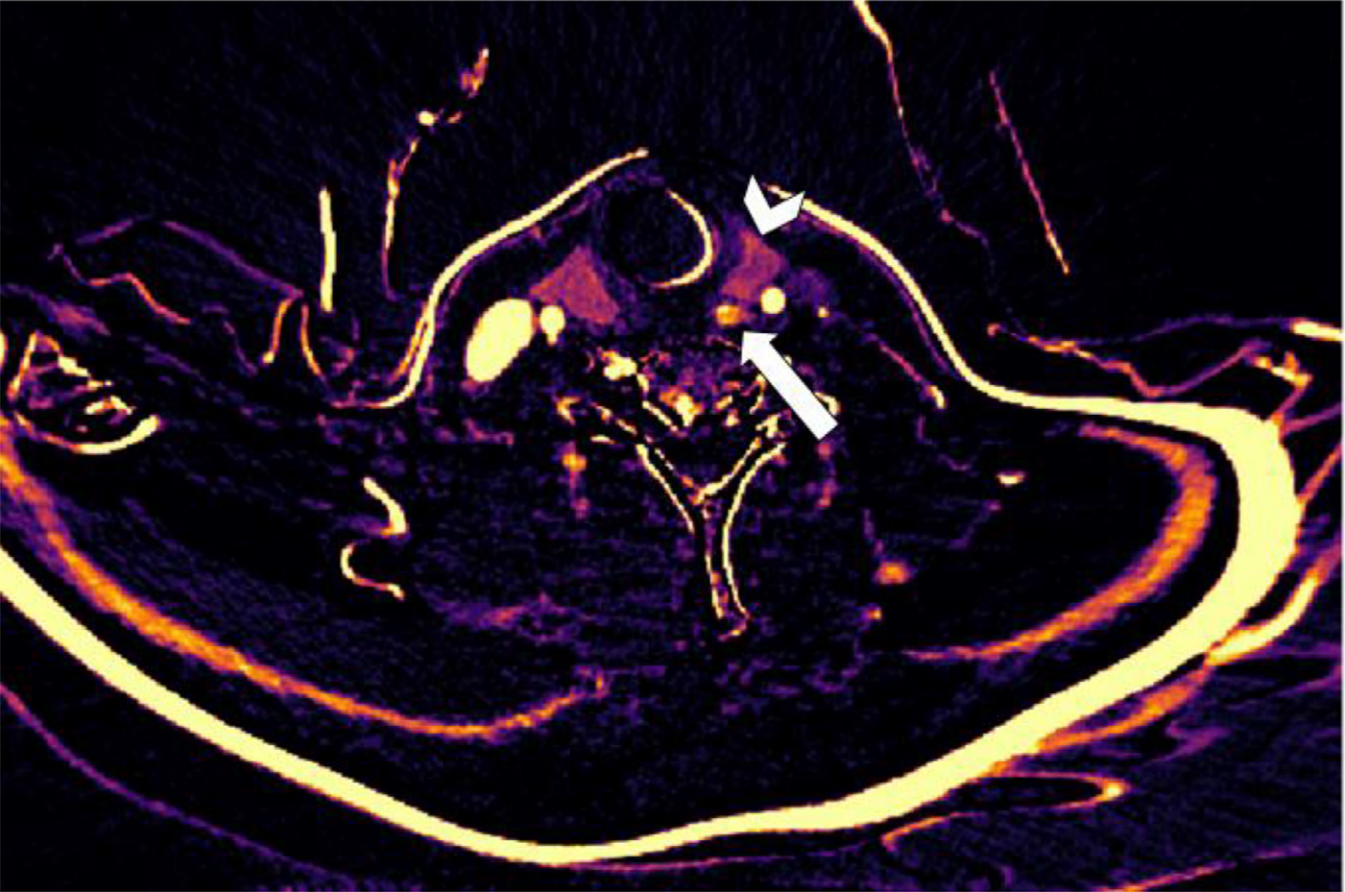
Subtraction map of patient 1. Arrowhead shows the thyroid gland. Straight arrow shows increased arterial enhancement in the first described lesion.

**Fig. 3:**
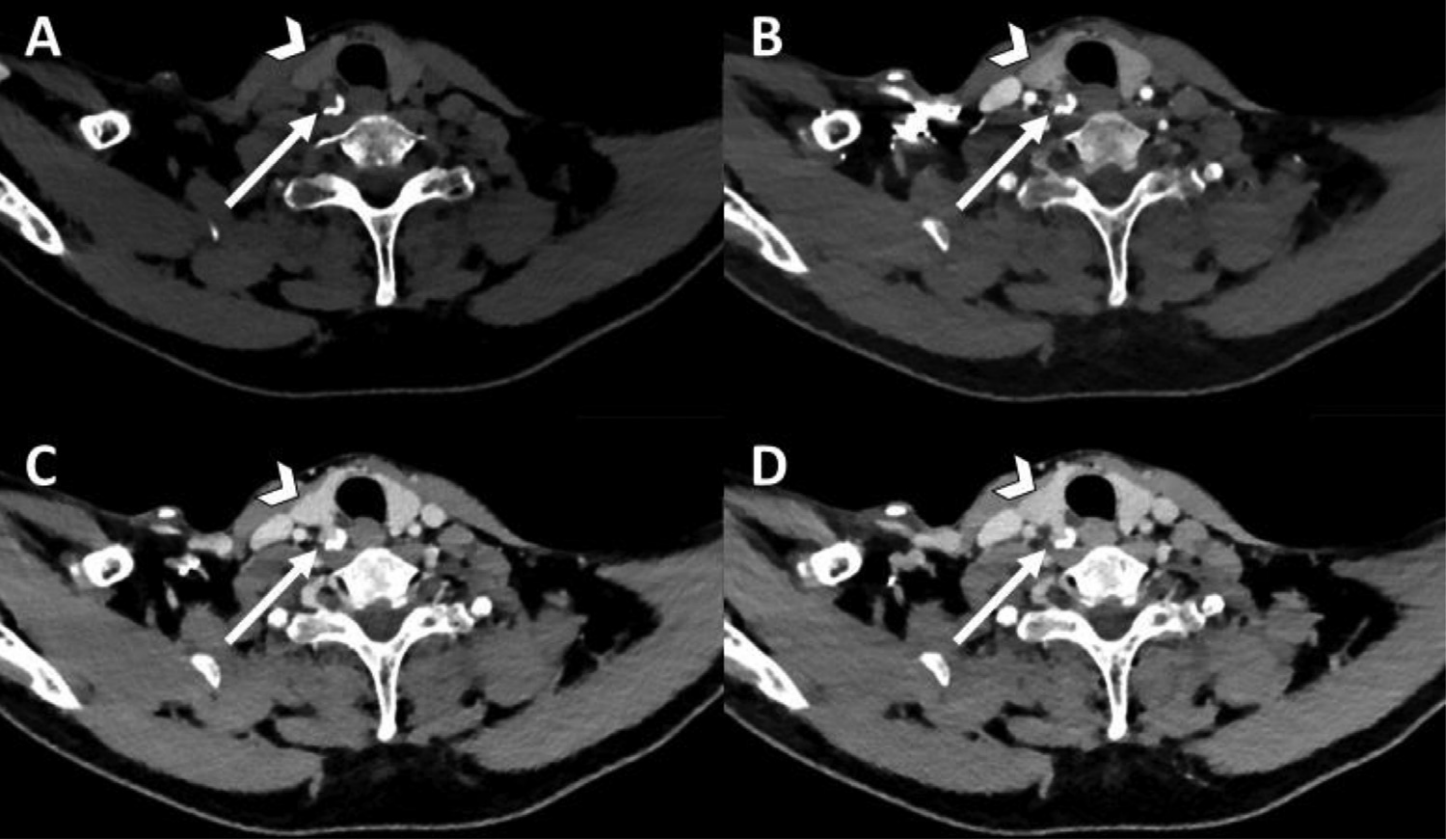
Transversal 4-dimensional CT with nonenhanced (A), arterial (B), venous (C) and delayed venous (D) phase of patient 1. The arrowhead is the thyroid gland. The arrow shows the partially calcified and cystic degenerated parathyroid adenoma, which was mistaken for a thyroid nodule. On the nonenhanced phase, it subtly shows that the noncalcified part shows lower attenuation than the thyroid gland, with arterial enhancement on the arterial phase. This was not reported at first, but after the subtraction map, the arterial enhancement was more clearly visible.

**Fig. 4:**
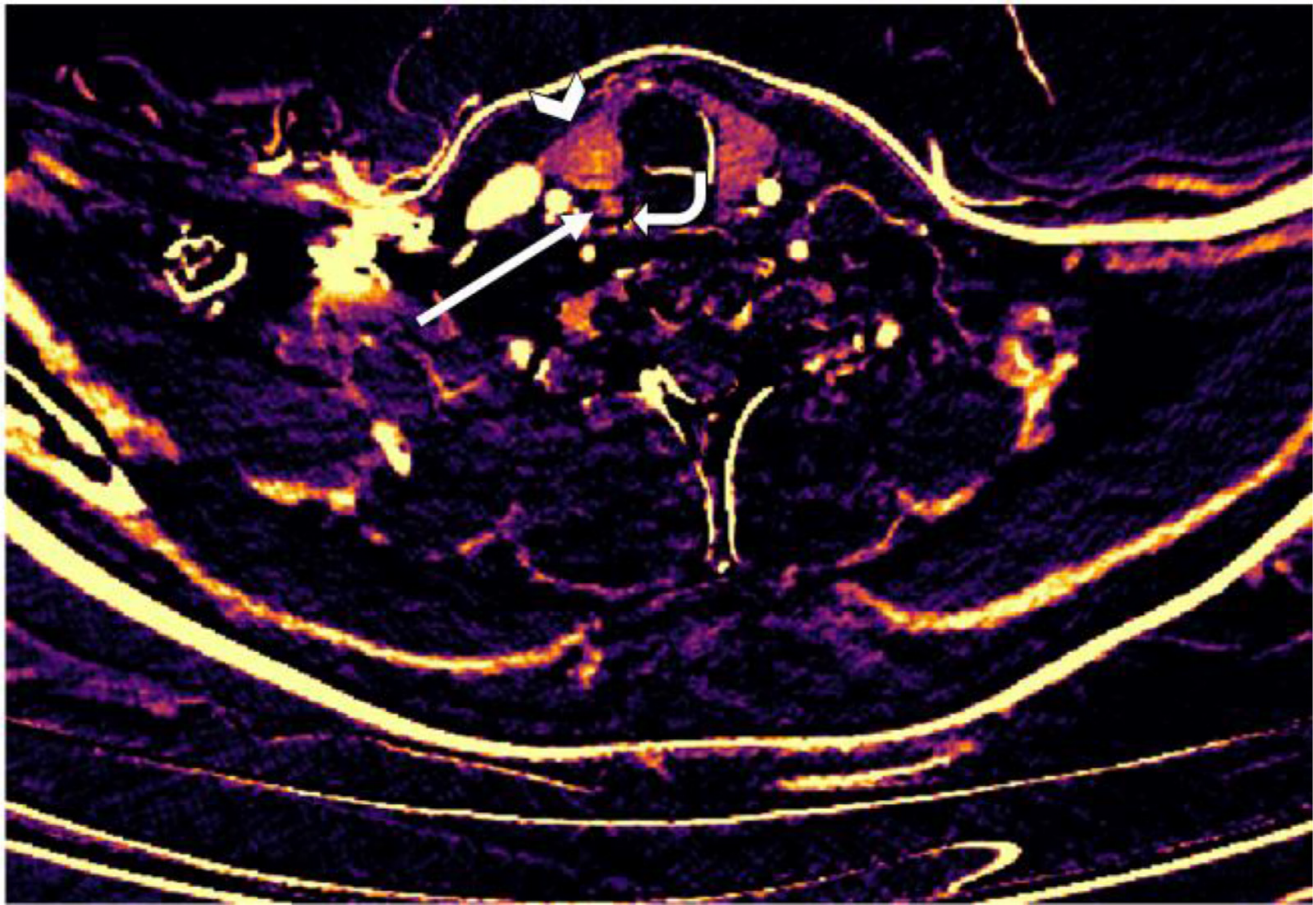
Subtraction map of patient 1. The arrowhead is the thyroid gland. The straight arrow shows increased arterial enhancement of the adenoma. The curved arrow shows the calcification without enhancement, making the enhancing part of the adenoma more detectable. Because of subtle movements between the series, there is a line of increased arterial attenuation artefact because of misalignment.

Patient 1 is a 54-year-old male with PHPT [PTH 45.3 pmol/L (normal range 2.0-9.3 pmol/L) and calcium 2.86 mmol/L (normal range 2.18-2.60 mmol/L)] and severe osteoporosis. Preoperative imaging consisted of a 4DCT on which a parathyroid adenoma was located dorsally of the left upper quadrant (see Fig. 1). A thyroid nodule was reported on the right side. During MIP, the preoperatively identified parathyroid adenoma on the left was removed. However, intraoperative PTH monitoring did not show an adequate decrease in PTH, so the neck was explored for a second adenoma. After excising a second adenoma on the right, PTH level dropped adequately within 40 minutes. When we retrospectively created the subtraction map, the first lesion showed increased arterial enhancement corresponding to the 4DCT (see Fig. 2). The lesion on the right, that was reported as thyroid nodule (see Fig. 3), showed increased arterial enhancement on the subtraction maps compared to the thyroid tissue as well (see Fig. 4). Histological assessment confirmed parathyroid adenoma both on the left and the right.

## Case 2

**Fig. 5:**
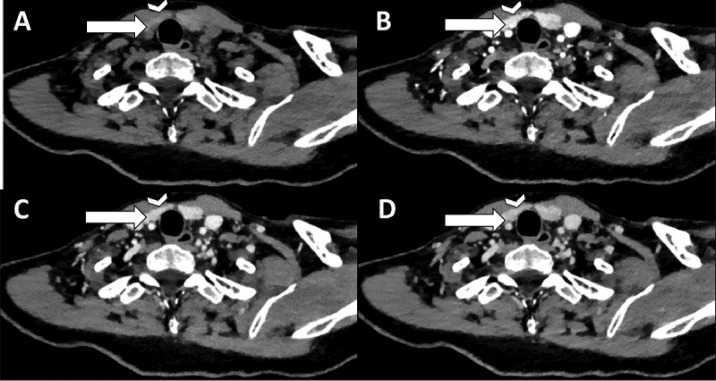
Transversal 4-dimensional CT with nonenhanced (A), arterial (B), venous (C) and delayed venous (D) phase of patient 2. The arrowhead is the thyroid gland. The arrow points to a small lesion with slight decreased attenuation on the nonenhanced phase compare to the thyroid gland, with arterial enhancement and subtle wash-out. This was not reported before the subtraction map was created.

**Fig. 6:**
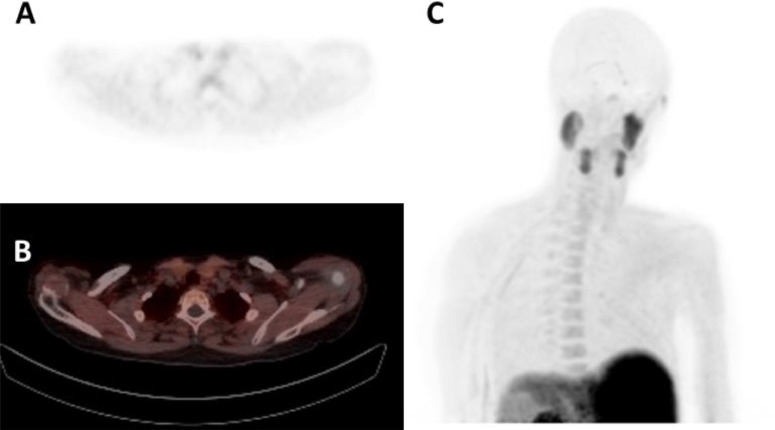
FCH-PET-CT of patient 2. Transversal PET (A) and fused PET-CT (B) images show no increased uptake at this lesion. The MIP (C) shows physiological FCH uptake, no increased uptake at the parathyroid glands.

**Fig. 7:**
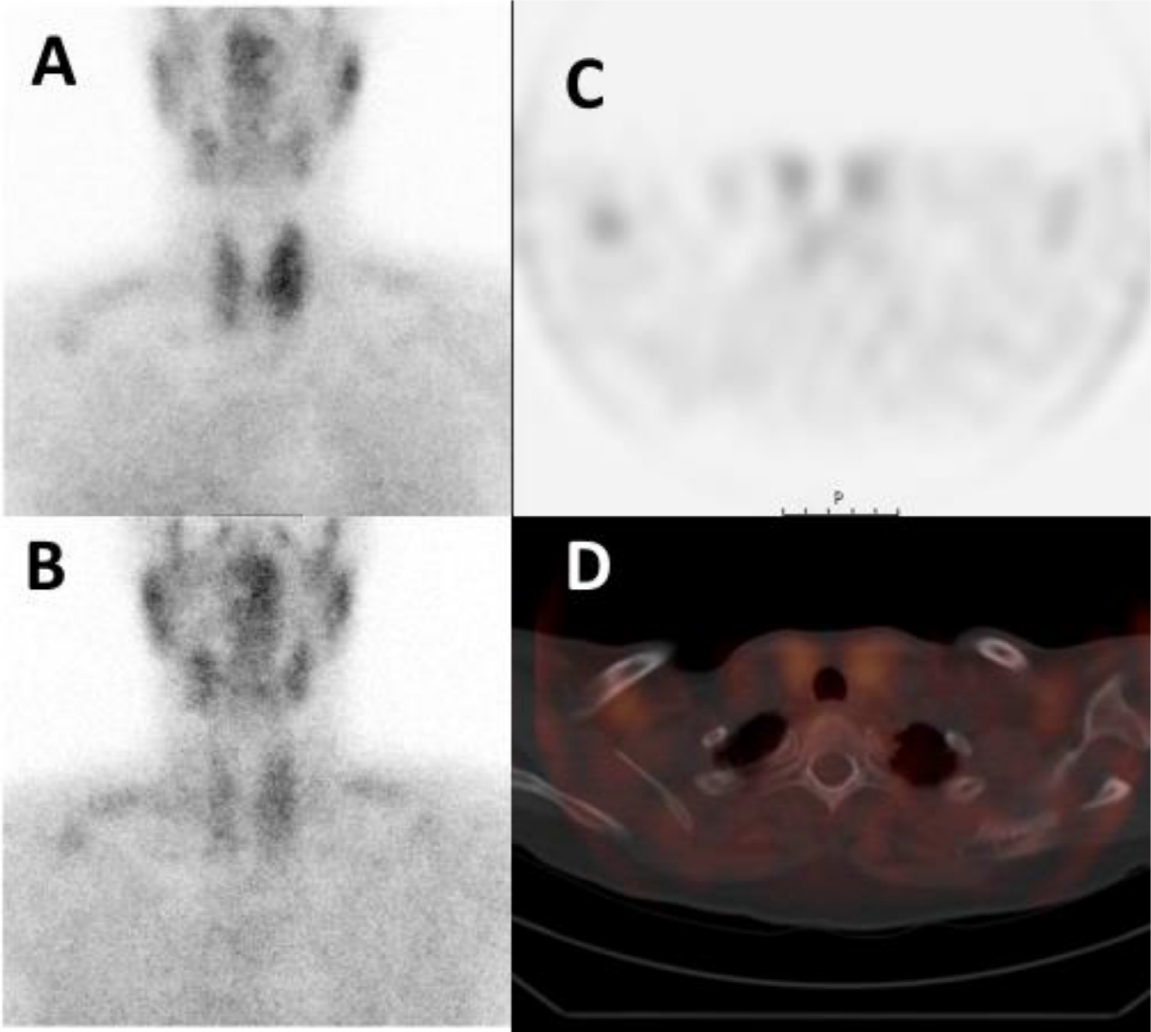
Tc99m-Sestamibi SPECT-CT of patient 2. No increased uptake was seen on the first phase (A) and no retention on the second phase planar images (B). Transversal SPECT (C) and fused SPECT-CT (D) image also show no increased Sestamibi uptake at this lesion.

**Fig. 8:**
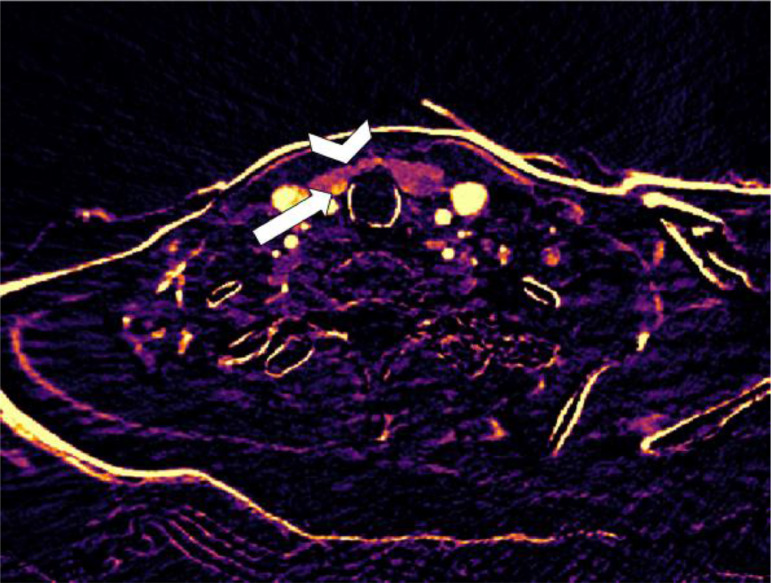
Subtraction map of patient 2. The arrowhead is the thyroid gland. The arrow points to a lesion with increased arterial enhancement just dorsally of the right thyroid gland lobe. This turned out to be the parathyroid adenoma after surgery.

The second patient is a 57-year-old female with PHPT (PTH 15.0 pmol/L and calcium 2.85 mmol/L) and osteopenia. A 4DCT, a Tc-99m-Sestamibi SPECT/CT and a F-18 choline (FCH)-PET/CT were acquired as preoperative imaging, all with negative result (Fig. 5, Fig. 6, Fig. 7). Subsequently, we have created a subtraction map, on which a parathyroid adenoma could be located since the lesion demonstrates a higher arterial contrast enhancement compared to the thyroid gland (Fig. 8). Using the information of the subtraction map, the parathyroid adenoma could be identified in retrospect on both the 4DCT and FCH-PET/CT as well, where the adenoma initially was missed. The treatment of this patient was changed from BNE to MIP.

## Case 3

**Fig. 9:**
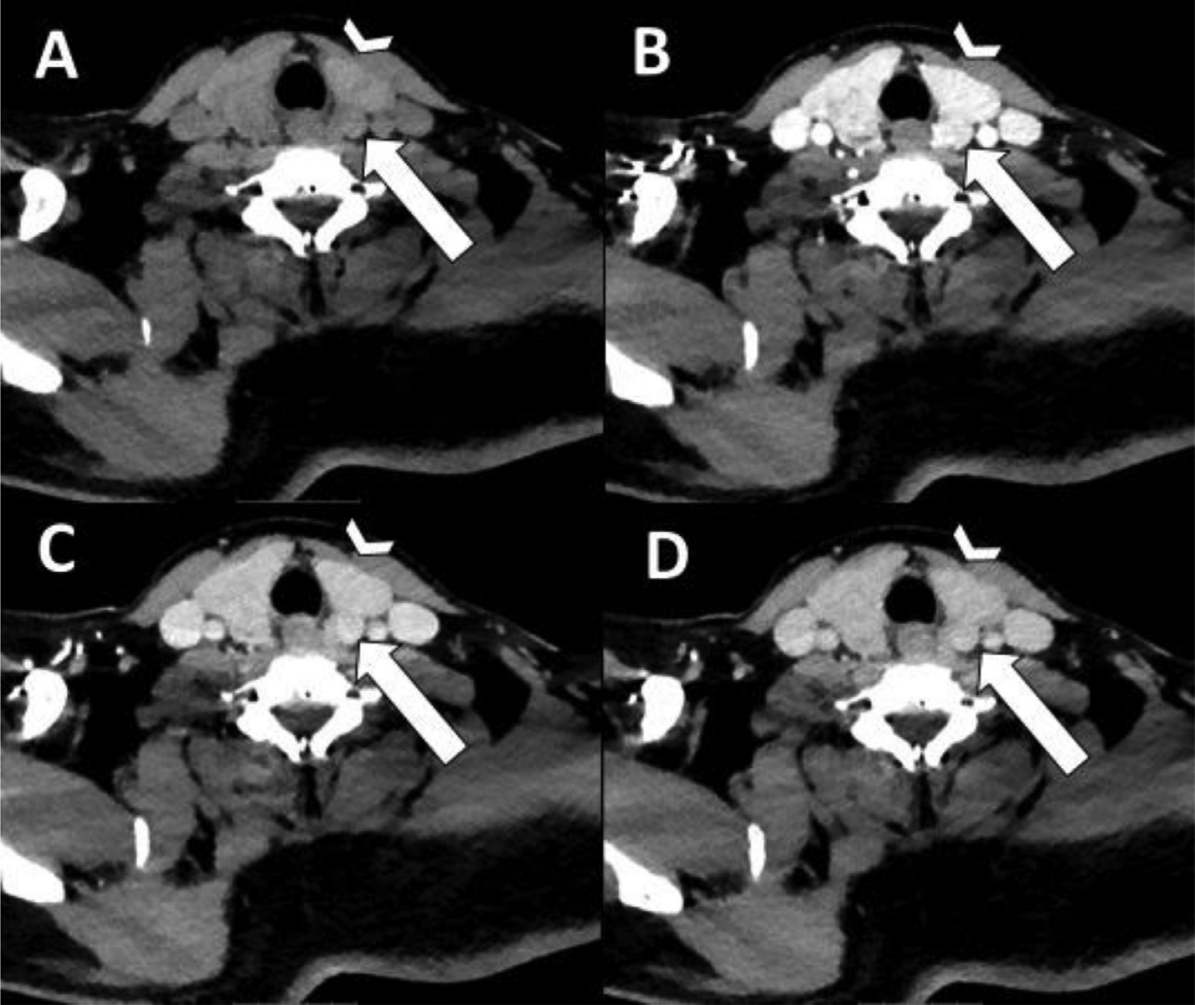
Transversal 4-dimensional CT with nonenhanced (A), arterial (B), venous (C) and delayed venous (D) phase of patient 3. The arrowhead is the thyroid gland. The straight arrow shows a lesion with subtle decreased attenuation on the nonenhanced phase compared to the thyroid gland on the left side. This lesion shows enhancement in the postcontrast phases comparable to the thyroid gland. There are also several thyroid nodules on the right, which makes it difficult to differentiate what the parathyroid adenoma could be.

**Fig. 10:**
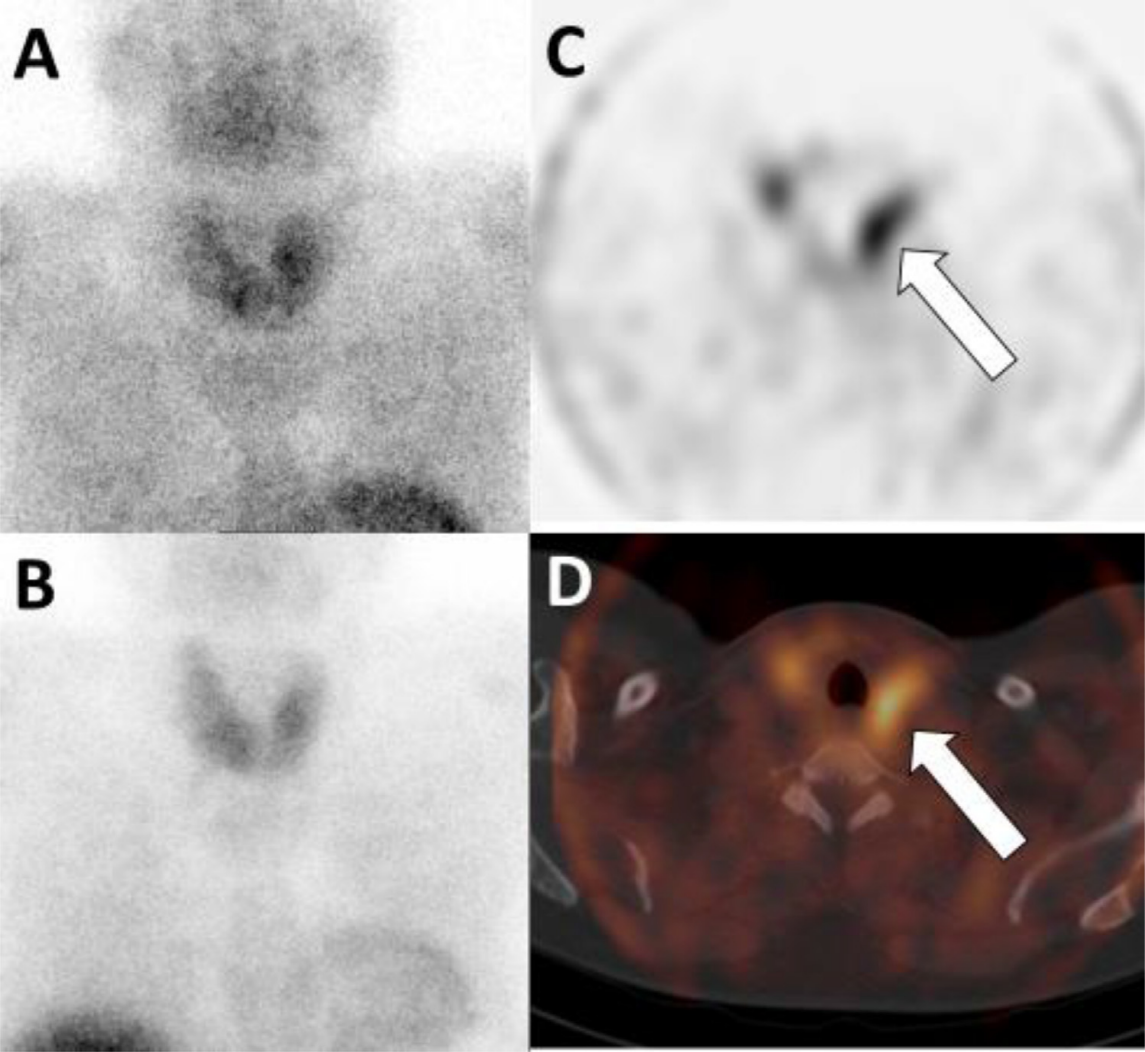
Tc99m –Sestamibi SPECT-CT of patient 3. On the first phase (A) increased uptake caudally on the right side and in the middle of the left thyroid lobe. The second phase (B) shows retention in both lesions. Transversal SPECT (C) and fused SPECT-CT (D) shows increased uptake dorsal from the left thyroid gland.

**Fig. 11:**
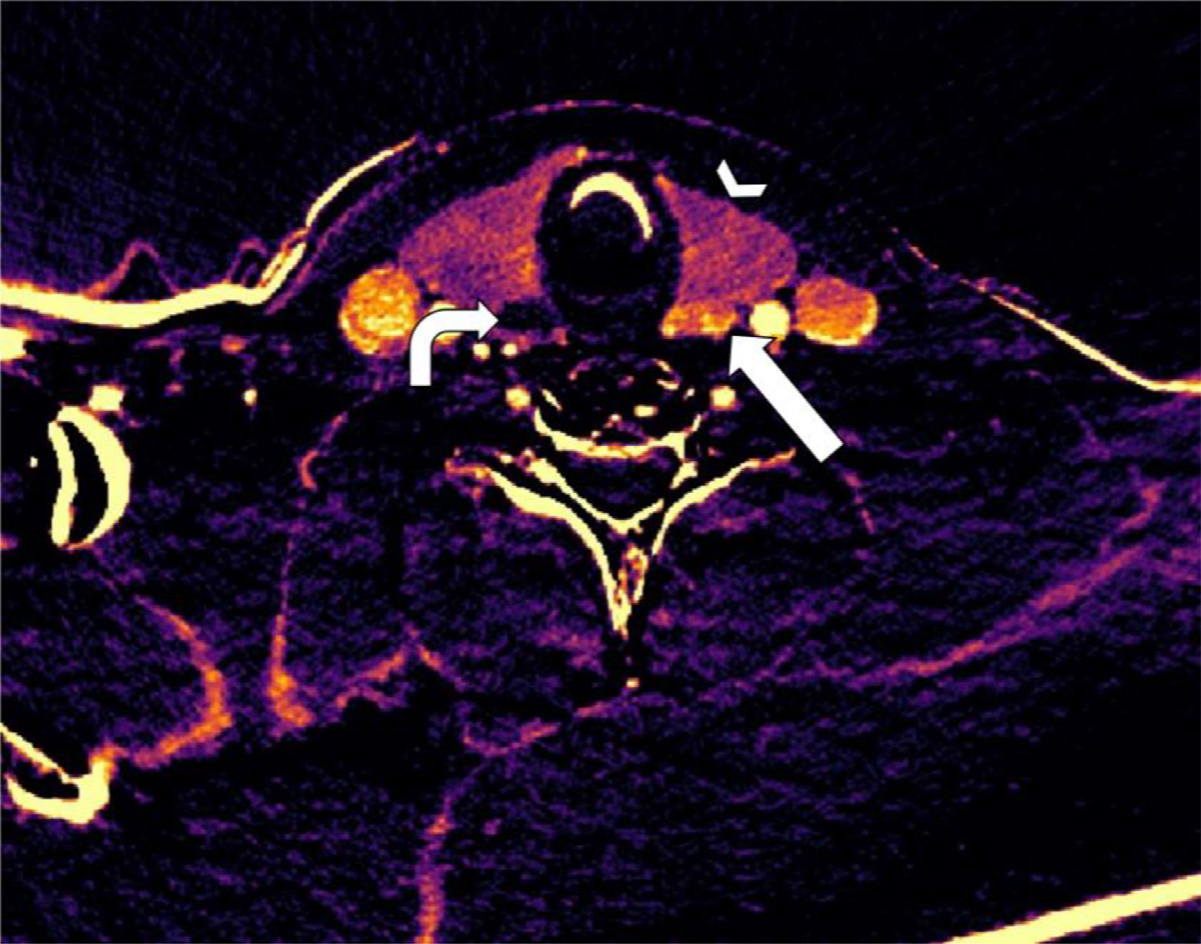
Subtraction map of patient 3 showing increased arterial enhancement of the lesion (straight arrow) dorsal from the left thyroid gland (arrowhead). The thyroid nodule on the right (curved arrow) shows no arterial enhancement, distinguishing it from the parathyroid lesion.

The third patient is a 51-year-old male with PHPT (PTH 17.6 pmol/L and calcium 2.82 mmol/L) and a vitamin D deficiency [29-47 nmol/L (normal range >50 nmol/L)]. A 4DCT was performed, on which no parathyroid adenoma was identified with certainty (Fig. 9). A subtraction map was created and showed a highly arterial enhancing lesion close to the left thyroid lobe (Fig. 11). Using this information, the adenoma was located on the nonenhanced and arterial phase series with more certainty. This location was confirmed by Tc-99m-Sestamibi SPECT/CT (Fig. 10).

## Discussion

The current principle to differentiate parathyroid adenomas from thyroid tissue is based on the rapid arterial contrast wash-in of parathyroid adenomas. Our subtraction map demonstrates the arterial enhancement of tissues, which is normally done by the radiologist by simultaneous visual evaluation of the different contrast phases, which is obviously subjective and highly dependent on experience. The subtraction map essentially automates this task and is therefore expected to increase the detection rate of parathyroid adenomas on 4DCT. In all 3 cases, the subtraction map was successful, as an adenoma was revealed that was not identified initially using the 4DCT.

There is a big variety in sensitivity in literature for 4DCT. Kluijfhout et al. [11] showed that sensitivity for 4DCT ranges from 48% to 92%. The subtraction map might increase sensitivity by showing the distinct arterial enhancement in 1 map instead of comparing the nonenhanced and arterial phase at the same time. The subtraction map may serve as a tool to detect lesions with increased arterial enhancement which can then be evaluated using the original series.

Two parathyroid adenomas were located on the subtraction map in our first case. In contrast, only 1 parathyroid adenoma was detected using 4DCT. Excision of this adenoma did not result in an adequate PTH decrease, while removal of the second adenoma did lead to satisfactory PTH reduction. This suggests superior evaluation of 4DCT when the subtraction map is used in addition to the original CT series, and it can be beneficial in case of possible double adenomas or multiglandular disease in particular. Moreover, the subtraction map may counter satisfaction of search, as the subtraction map clearly reveals possible structures that show increased arterial enhancement, which could improve the detection rate for double adenomas or multiglandular disease.

Furthermore, the first case demonstrates that the subtraction map might be superior in differentiation between thyroid nodules and parathyroid adenomas in close relation to the thyroid. In our case, the cystic parathyroid adenoma was mistakenly regarded as cystic thyroid tissue, based on the 4DCT. During surgery, there was no adequate PTH decrease after excising the adenoma on the left and a second adenoma was identified, which was subsequently confirmed by histology. In retrospect, we have created a subtraction map which revealed a clear difference in arterial contrast enhancement between the parathyroid adenoma and the thyroid. This suggests that the addition of a subtraction map may remarkably improve differentiation of cystic adenomas and/or adenomas located closely to the thyroid.

The second case shows that a subtraction map might increase detection rates for 4DCT. In this case, the adenoma was located on the subtraction map, while being undetected on other imaging modalities. Because of the subtraction map, the parathyroid adenoma was identified, which in retrospect could then be located on the 4DCT and FCH-PET/CT as well. As a result, the surgery changed from the invasive intervention BNE to MIP.

The third case shows using the subtraction map as confirmation tool. Initially, an experienced radiologist could not identify the adenoma with certainty. The subtraction scan confirmed the expected location, which was later confirmed by the SPECT/CT. Therefore, no additional imaging was necessary.

## Conclusion

This report of 3 cases shows the potential benefit of a subtraction map in addition to the 4DCT. It is a convenient tool for sensitive detection of possible double adenomas or multiglandular disease. Further research is necessary to determine the role of subtraction maps in 4DCT and preoperative imaging for PHPT.

## Patient consent

Written informed consent for the publication of this report of 3 cases was obtained from the patients.
